# Laparoscopic Hepatectomy: Current State in Japan Based on the 4th Nationwide Questionnaire

**DOI:** 10.1155/2017/6868745

**Published:** 2017-03-12

**Authors:** Yutaka Takahashi, Satoshi Katagiri, Shun-ichi Ariizumi, Yoshihito Kotera, Hiroto Egawa, Go Wakabayashi, Hironori Kaneko, Masakazu Yamamoto

**Affiliations:** ^1^Department of Surgery, Institute of Gastroenterology, Tokyo Women's Medical University, 8-1 Kawada-cho, Shinjuku-ku, Tokyo, Japan; ^2^Department of Surgery, Ageo Central General Hospital, 1-10-10 Kashiwaza, Ageo-shi, Saitama, Japan; ^3^Division of General and Gastroenterological Surgery, Department of Surgery (Omori), Toho University School of Medicine, 6-11-1 Ohmori-nishi, Ohta-ku, Tokyo, Japan

## Abstract

*Purpose.* Since laparoscopic hepatectomy (LH) became covered by national health insurance in April 2010 in Japan, the numbers of applied cases and institutions performing it have increased and the indication has expanded. We surveyed the current state and safety of LH in Japan. *Methods.* A questionnaire survey was performed in 41 institutions related to the Japanese Endoscopic Liver Surgery Study Group and 747 institutions certified by the Japanese Society of Gastroenterological Surgery, and responses concerning all 2962 cases of LH performed by August 2011 were obtained. *Results.* The surgical procedure employed was hemihepatectomy in 234 (8%), segmentectomy in 88 (3%), left lateral segmentectomy in 434 (15%), segmentectomy in 156 (5%), and partial resection in 1504 (51%) cases. The approach was pure laparoscopy in 1835 (63%), hand-assisted laparoscopic surgery in 201 (7%), and laparoscopy-assisted surgery in 926 (31%). Regarding perioperative complications, surgery was switched to laparotomy in 59 (2.0%), reoperation was performed in 4 (0.1%), and surgery-related death occurred in 2 (0.07%). Intraoperative accidents occurred in 68 (2.3%), and postoperative complications developed in 94 (3.2%). *Conclusions.* When the selection of cases is appropriate, LH for liver diseases can be safely performed.

## 1. Introduction

Laparoscopic hepatectomy (LH) was initially performed to resect a benign liver tumor by Reich et al. in 1991 [1]. LH requires a large incision to mobilize the liver in addition to the resection of the liver parenchyma. Moreover, the liver is surrounded by the costal bones, and securing a visual field is difficult. Performing these procedures in the abdominal cavity under laparoscopy reduces the size of the incision and burden on the patient [2]. Based on this concept, LH has recently spread rapidly throughout the world, and now, it is applied for not only surgery of malignant tumors but also donor surgery in living donor liver transplantation. The procedures of LH were already established and reported by institutions whose staff had encountered many cases overseas [3]. An international consensus meeting was held in Louisville in November 2008, in which the surgical procedure and safety of LH were widely discussed and the guidance for the development of the current LH was presented [4].

In Japan, since LH was initially applied for liver cancer by Hashizume et al. in 1995 and Kaneko et al. in 1996, cases resected by LH have occasionally been reported [5, 6]. In April 2010, partial resection and lateral segmentectomy became covered by national health insurance, and the numbers of cases and institutions performing LH are rapidly increasing. The current state of LH in Japan has been surveyed every year by the Japanese Endoscopic Liver Surgery Study Group since 2007 [7–9]. The 4th Annual Meeting of the Japanese Endoscopic Liver Surgery Study Group (chairman: M. Yamamoto) was held in November 2010. This questionnaire survey was performed by this Study Group to investigate the current state of LH in Japan and its safety. We report the results of this survey.

## 2. Methods

The questionnaire survey has been performed by the Japanese Endoscopic Liver Surgery Study Group since 2009, and this was the 4th survey. The questionnaire was sent by post mail to 41 institutions related to the Study Group and 747 institutions certified by the Japanese Society of Gastroenterological Surgery. The subjects of the survey were all patients who underwent the first laparoscopic hepatectomy at the institutions by August 31, 2011.

## 3. Results

Four hundred sixty-four institutions (59%) responded to the questionnaire, 113 (14.3%) of these perform LH, and the total number of cases was 2962.

**Figure 1:**
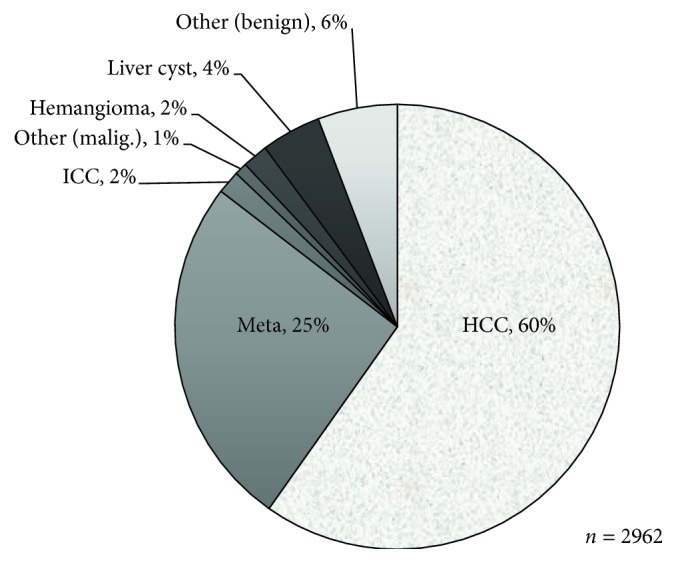
The treated disease.

**Figure 2:**
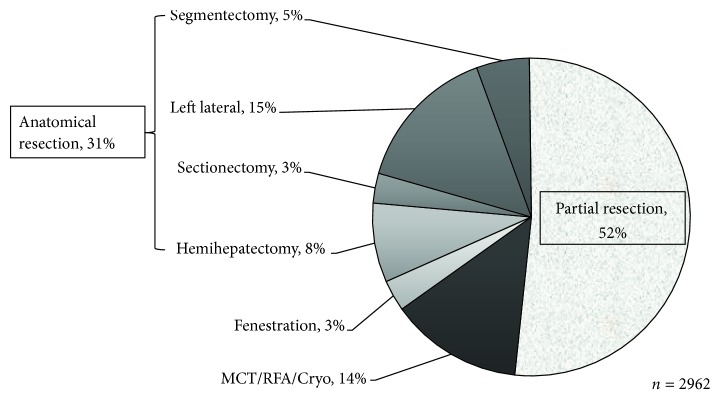
Surgical procedures for laparoscopic hepatectomy.

**Figure 3:**
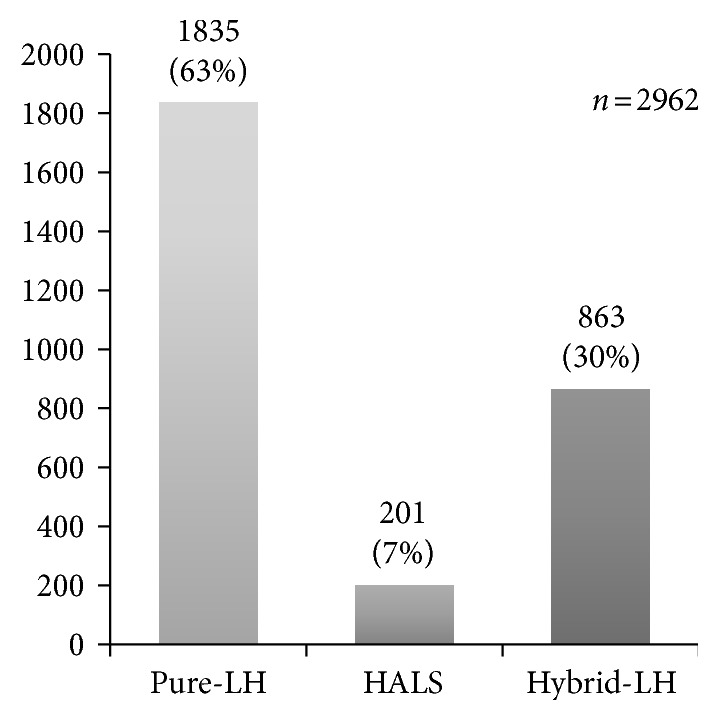
Type of surgical approach.

The treated disease was hepatocellular carcinoma in 1840 cases (62%), metastatic liver cancer in 762 (26%), intrahepatic cholangiocarcinoma in 50 (2%), other malignant tumors in 27 (1%), hemangioma in 55 (2%), liver cyst in 129 (4%), and other benign diseases in 172 (6%) (Figure 1). The surgical procedure was hemihepatectomy in 234 (8%), sectionectomy (excluding left lateral segmentectomy) in 88 (3%), left lateral segmentectomy in 434 (15%), segmentectomy in 156 (5%), partial resection in 1504 cases (51%), MCT/RFA in 454 (15%), and fenestration in 92 (3%) (Figure 2). The laparoscopic approach was complete laparoscopy (Pure-LH) in 1835 (63%), hand-assisted laparoscopic surgery (HALS) in 201 (7%), and laparoscopy-assisted surgery (Hybrid-LH) in 926 (31%) (Figure 3). By the approach, systematic and partial resections were performed in 912 (31%) and 1504 (51%) in all cases, 398 (22%) and 986 (54%) in Pure-LH cases, 42 (21%) and 129 (64%) in HALS cases, and 472 (55%) and 389 (45%) in Hybrid-LH cases, respectively (Table 1). When the cases treated with each surgical procedure employing each approach were divided into HCC and Meta, systematic and partial resections were applied to HCC by Pure-LH in 240 (21%) and 595 (51%) cases and by Hybrid-LH to 286 (48%) and 244 (41%), respectively. In Meta cases, systematic and partial resections were applied by Pure-LH to 114 (26%) and 303 (69%) and by Hybrid-LH to 76 (38%) and 124 (61%), respectively (Table 1).

The surgical procedure of systematic resection was unilateral lobectomy in 234 (26%), and the approach employed for this unilateral lobectomy was Hybrid-LH in 187 (37%) and Pure-LH in 41 (18%). The number of cases treated with lateral segmentectomy was 434 (48%), being the largest, and the approach was Hybrid-LH in 259 cases (60%). By the disease (HCC and Meta), systematic resection of HCC was performed employing unilateral lobectomy in 91 (16%), segmentectomy in 347 (63%) (including 286 cases (52%) of lateral segmentectomy), and subsegmentectomy in 110 (20%). Of the 286 cases of lateral segmentectomy-treated HCC, the approaches were Pure-LH and Hybrid-LH in 150 (52%) and 122 (43%), respectively. Of the 92 lateral segmentectomy-treated Meta cases, Pure-LH and Hybrid-LH were employed in 77 (84%) and 6 (7%), respectively (Table 2).

Partial resection was performed employing Pure-LH in 986 cases (66%), HALS in 129 (9%), and Hybrid-LH in 389 (26%). The resected region was S6 in 374 (25%), S3 in 315 (20.9%), and S5 in 204 (14%) (Table 3).

Regarding perioperative complications, the procedure was switched to laparotomy in 59 cases (2.0%), reoperation was performed in 4 (0.1%), and surgery-related death occurred in 2 (0.07%). Intraoperative accidents occurred in 68 (2.3%) (hemorrhage in 39 (1.3%) and hypercapnia in 1 (0.03%)). Postoperative complications developed in 94 (3.2%) (hemorrhage in 8 (0.3%), liver failure in 2 (0.07%), bile leakage in 15 (0.5%), thoracicoabdominal fluid in 24 (0.8%), wound infection in 18 (0.6%), liver abscess in 9 (0.3%), hypercapnia in 1 (0.03%), and ileus in 2 (0.07%)) (Table 4). By the approach, the procedure was switched to laparotomy from Pure-LH in 41 cases (2.2%), from HALS in 12 (6.0%), and from Hybrid-LH in 6 (0.8%). Intraoperative accidents occurred under Pure-LH in 54 (2.9%), HALS in 6 (3.0%), and Hybrid-LH in 8 (0.9%), and postoperative complications developed after Pure-LH in 34 (1.9%), HALS in 12 (6.0%), and Hybrid-LH in 48 (5.6%) (Table 4).

**Figure 4:**
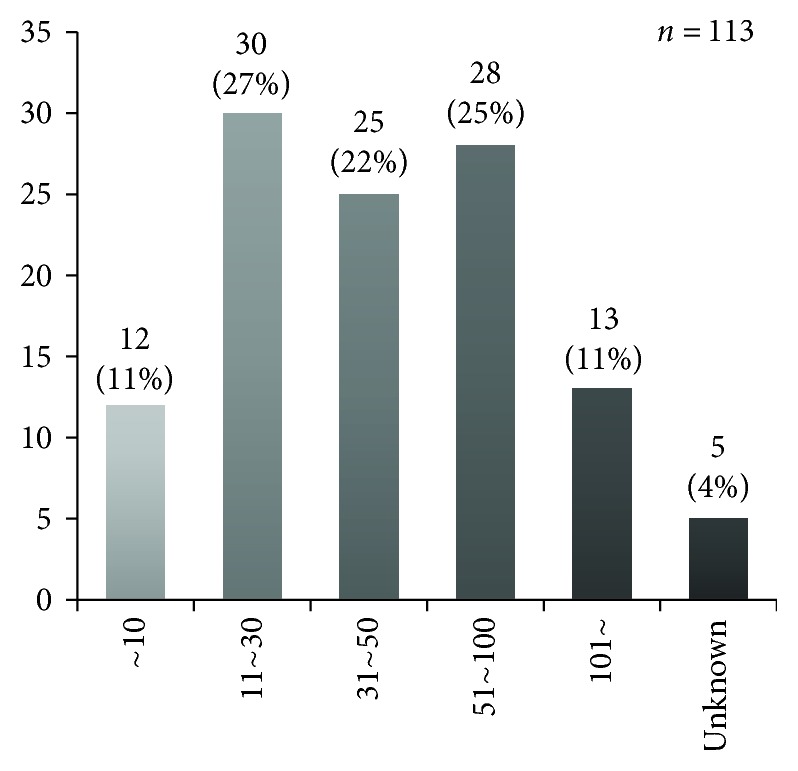
The number of part-year hepatectomy (open and laparoscopic) cases in each institute.

Of the 113 institutions performing laparoscopic hepatectomy which responded to the questionnaire, the annual number of hepatectomy-treated cases (including laparotomy) was 101 or more in 13 institutions (11%), 51–100 in 28 (25%), 31–50 in 25 (22%), 11–30 in 30 (27%), and 10 or fewer in 12 (11%) (Figure 4).

**Figure 5:**
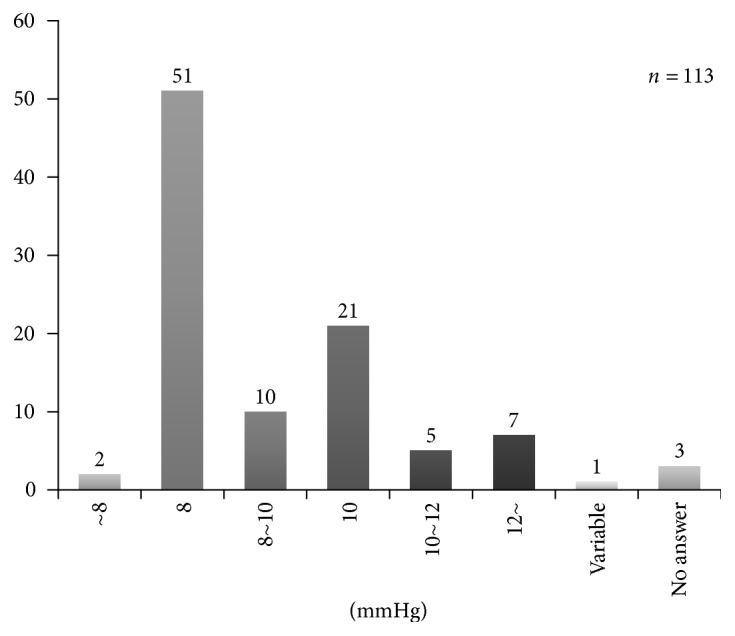
The number of institute on each intraoperative pneumoperitoneum pressure.

The intraoperative pneumoperitoneum pressure was 12 mmHg or higher in 8 institutions (7%), 10–12 mmHg in 29 (26%), 8–10 mmHg in 71 (63%), and lower than 8 mmHg in 2 (2%) (Figure 5).

## 4. Discussion

LH was partially covered by national health insurance in April 2010 in Japan, and partial resection and lateral segmentectomy are covered, in which the numbers of cases and institutions performing these may be increasing. The questionnaire survey has been performed since 2009 when the Japanese Endoscopic Liver Surgery Study Group was established, and this was the 4th survey. The first and second surveys were performed in institutions related to the study group [7, 8], but institutions certified by the Japanese Society of Gastroenterological Surgery were additionally included from the previous 3rd survey [9]. Of the 464 institutions (59%) which responded, 113 (14.3%) performed LH, and the total number of LH-resected cases increased by 640 to 2899 per year, although the number of responder institutions decreased. This increase may have been due to the marked influence of coverage by national health insurance one year and 4 months ago.

HCC and Meta cases accounted for 60 and 25%, respectively, and HCC cases decreased and Meta cases increased from those in the previous survey [9] (HCC: 64.9%, Meta: 20.0%). The approach was Pure-LH in 59.6% and Hybrid-LH in 32.7% in the previous survey [9], and cases employing Pure-LH slightly increased and those employing Hybrid-LH decreased. The surgical procedure was partial resection in 52%, lateral segmentectomy in 15%, subsegmentectomy in 5%, and segmentectomy (excluding lateral segmentectomy) in 3%, showing that partial resection and lateral segmentectomy covered by national health insurance accounted for 67% of all cases. Cases treated with lateral segmentectomy and unilateral lobectomy increased, and those treated with partial resection decreased compared to those in the previous year. The surgical procedure and approach tended to shift from Hybrid-LH to Pure-LH and from partial to systematic resection, respectively. LH advanced with the improvement of operators' techniques and progression of various devices. As of several years after coverage by national health insurance, the installation of various devices may have progressed with the improvement of operators' techniques. In the future, the number of LH cases may rapidly increase and the shifts from Hybrid-LH to Pure-LH and from partial to systematic resection may become marked.

When the surgical procedure was investigated by the approach, while Pure-LH and Hybrid-LH were employed in 1835 (63%) and 863 (30%) of all cases, respectively, these were employed in 398 (43.6%) and 472 (51.8%) cases of systematic resection and 986 (65.6%) and 389 (25.9%) of partial resection, respectively, showing that Hybrid-LH tended to be selected for systematic resection and Pure-LH tend to be selected for partial resection (Table 1).

When systematic resection employing each surgical procedure was analyzed by the approach in HCC and Meta cases (Table 2), while unilateral lobectomy was performed in 26% of all cases, it was performed in only 16% of HCC cases and 28% of Meta cases, which is nearly the same rate in all cases. Lateral segmentectomy was performed under Pure-LH in 259 (59.7%) and Hybrid-LH in 151 (34.8%) of all cases. It was performed under Pure-LH and Hybrid-LH in 150 (52.4%) and 122 (42.7%) of HCC cases and 77 (83.7%) and 6 (6.5%) of Meta cases, respectively, suggesting that major resection is tended to be avoided in HCC cases because of the risk of hemorrhage due to the presence of chronic hepatitis and background liver cirrhosis and due to the selection of small-range resection to conserve the residual liver function. Since unilateral lobectomy is not necessarily technically more advanced than subsegmentectomy, a high rate of employing segmentectomy and subsegmentectomy may continue to conserve the residual liver function, even though techniques will progress in the future.

Partial resection was performed under complete laparoscopy in 986 cases (66%), and this rate did not change in the HCC and Meta cases. The most frequently resected region was S6 (374 cases, 25%) followed by S3 (315 cases, 20.9%) and S5 (204 cases, 14%). When these cases were divided into HCC and Meta, the most frequently resected region was S6 followed by S3 and S5, showing the same order. S6, S3, and S5 are located in the marginal region on the liver foot side considered to be relatively easy to resect, and the findings were as expected. On the other hand, partial resections of S7 and S8 on the dorsal liver head side are considered relatively difficult to resect, but these were performed in 109 (7%) and 167 (11%) cases, respectively, not being markedly low. S1 was resected in 12 (0.8%), showing the lowest rate. Although this region is not necessarily difficult to resect, its resection tended to be avoided, possibly because the visual field is restricted (Table 3).

Regarding perioperative complications, the procedure was switched to laparotomy in 59 (2.0%), reoperation was performed in 4 (0.1%), and surgery-related death occurred in 2 (0.07%). No marked change from those in the previous survey [9] was noted. The frequency of switching to laparotomy of 2% was lower (0.66–8.1%, mean: 4.9%) [10] than that of laparoscopic cholecystectomy, which is widely performed with a standardized technique in Japan. The most commonly expressed reason is that we have an established system of endoscopic surgical skill qualification by the Japan Society for Endoscopic Surgery. And we have an institute authorization system of laparoscopic hepatectomy on covering by insurance. Therefore, we believe that surgical skill can be kept at a high level due to these credentialing and authorization systems. Another reason may be a problem with the definition of switching to laparotomy. For example, when hemorrhage from the transected liver surface cannot be controlled in Pure-LH and laparotomy is added to stop the bleeding, opinions differ on whether it is regarded as switching to laparotomy or Hybrid-LH. One more reason may be inaccurate data extraction from the database of each institution to the respond to the questionnaire, which is a disadvantage of questionnaire surveys. It is possible that complications and accidents are not accurately reported. It is necessary to establish accurate databases in cooperation with the Japan Surgical Society-based database, the National Clinical Database (NCD).

The intraoperative pneumoperitoneum pressure was 12 mmHg or higher in 8 institutions (7%), 10–12 mmHg in 29 (26%), 8–10 mmHg in 71 (63%), and lower than 8 mmHg in 2 (2%). Attention should be paid to it even in laparoscopic hepatectomy because gas embolism by carbon dioxide entering through the hepatic vein is problematic, but carbon dioxide embolism as an intraoperative accident has been reported to be rare in Western countries [11]. Complication by gas embolism occurred in 2 cases in the survey, and the incidence was 0.06%, being very rare. In animal experiments, gas embolism in the heart can be observed by transesophageal echocardiography in all animals treated with LH [12, 13]. In an experiment in which LH was performed at various central venous pressures (CVP), gas embolism affecting the circulatory dynamics was observed only in the low CVP group [14]. Control of the pneumoperitoneum pressure and CVP by consulting with an anesthesiologist may prevent serious gas embolism. Control of the pneumoperitoneum pressure is also useful for hemostasis, which is a merit of LH, in addition to the prevention of gas embolism. However, an excess pneumoperitoneum pressure may cause gas embolism, to which attention should be paid, and adjustment while judging the circulatory dynamics and grade of hemorrhage is necessary. Bile leakage as a postoperative complication occurred in 15 cases (0.05%). Transection in LH largely depends on an energy device, and delayed bile leakage due to deep cauterization conducted more than necessary is of concern [15], but the incidence is not high compared to that after laparotomy [16].

The mortality was as low as that in the previous survey, and it was much lower than that in Western countries [17]. Not all episodes may have been accurately reported, as described above, and there may have been more fatal cases. Close investigation using the NCD database is necessary.

## Figures and Tables

**Table 1:** Surgical procedures in each surgical approach (HCC/Meta).

	Pure-LH	HALS	Hybrid-LH	Total
Anatomical resection	**398** 240/114	**42** 22/17	**472** 286/76	**912** 548/207
Partial resection	**986** 595/303	**129** 65/47	**389** 244/124	**1504** 904/474
MCT/RFA/Cryo	**359** 334/22	**30** 8/22	**65** 63/2	**454** 405/46
Fenestration	**92** 0/0	**0** 0/0	**0** 0/0	**92** 0/0
Total	**1835** 1169/439	**201** 95/86	**926** 593/202	**2962** 1857/727

**Table 2:** Anatomical resection (all/HCC/Meta).

			Pure-LH		HALS		Hybrid-LH		Total
Hemihepatectomy	Right		**19**/7/9		**4**/1/2		**87**/39/16		**110**/47/27 (12/8/13%)
	Left		**22**/10/6		**2**/1/0		**100**/33/25		**124**/44/31 (14/8/15%)

Sectionectomy	Left lateral		**259**/150/77		**24**/14/9		**151**/122/6		**434**/286/92 (48/52/44%)
	Anterior		**8**/7/1		**0**/0/0		**20**/7/11		**28**/14/12 (3/3/6%)
	Posterior		**17**/13/4		**1**/0/1		**23**/20/2		**41**/33/7 (4/6/3%)
	Other		**7**/7/0		**0**/0/0		**12**/7/4		**19**/14/4 (2/3/2%)

Segmentectomy	S1	S2	**1**/1/0	**3**/1/2	**1**/0/1	**0**/0/0	**0**/0/0	**1**/1/0	**156**/110/34 (17/20/16%)
	S3	S4	**13**/8/5	**8**/6/1	**4**/1/1	**0**/0/0	**3**/3/0	**13**/7/2	
	S5	S6	**15**/12/2	**16**/11/5	**0**/0/0	**4**/3/1	**20**/14/3	**28**/26/1	
	S7	S8	**4**/3/1	**6**/4/1	**1**/1/0	**1**/1/0	**1**/1/0	**13**/7/6	

Total			**398**/240/114 (44/44/55%)		**42**/22/17 (5/4/8%)		**472**/286/76 (52/52/37%)		**912**/548/207

**Table 3:** The number of partial resection cases in each segment (all/HCC/Meta).

	Pure-LH	HALS	Hybrid-LH	Total
S1	**10**/6/4	**0**/0/0	**2**/1/1	**12**/7/5 (0.8/0.7/1%)
S2	**107**/65/19	**16**/14/2	**29**/22/3	**152**/101/24 (10/11/5%)
S3	**232**/139/60	**14**/9/3	**69**/41/21	**315**/189/84 (21/21/18%)
S4	**122**/69/45	**4**/1/3	**45**/34/10	**171**/104/58 (11/12/12%)
S5	**149**/85/52	**19**/8/5	**36**/18/15	**204**/111/72 (14/12/15%)
S6	**216**/143/65	**45**/22/14	**113**/78/33	**374**/243/112 (25/27/24%)
S7	**61**/33/26	**17**/5/12	**31**/17/12	**109**/55/50 (7/6/11%)
S8	**89**/55/32	**14**/6/8	**64**/33/29	**167**/94/69 (11/10/15%)
Total	**986**/595/303 (66/66/64%)	**129**/65/47 (9/7/10%)	**389**/244/124 (26/27/26%)	**1504**/904/474

**Table 4:** The rate of conversion to open surgery and complications in each surgical approach.

	All (*n* = 2899)	Pure-LH (*n* = 1835)	HALS (*n* = 201)	Hybrid-LH (*n* = 863)	HCC (*n* = 1857)	Meta (*n* = 727)
Conversion to open surgery	**59** (2.0%)	**41** (2.2%)	**12** (6.0%)	**6** (0.6%)	**49** (2.6%)	**8** (1.1%)
Mortality	**2** (0.07%)	**1** (0.05%)	**0**	**1** (0.01%)	**1** (0.05%)	**1** (0.13%)
Reoperation	**4** (0.14%)	**3** (0.16%)	**0**	**1** (0.01%)	**3** (0.16%)	**1** (0.13%)

Intraoperative accidents	**68** (2.3%)	**54** (2.2%)	**6** (3.0%)	**8** (0.9%)	**55** (2.9%)	**9** (1.2%)
Bleeding	39 (1.3%)					
Hyper carbon dioxidemia	1 (0.03%)					
Gas embolism	2 (0.06%)					

Postoperative complications	**94** (3.2%)	**34** (1.9%)	**12** (6.0%)	**48** (5.6%)	**66** (3.5%)	**24** (3.3%)
Bleeding	8 (0.3%)					
Liver failure	2 (0.07%)					
Bile leakage	15 (0.5%)					
Pleural effusion/ascites	24 (0.8%)					
Surgical site infection	18 (0.6%)					
Liver abscess	9 (0.3%)					
Hyper carbon dioxidemia	1 (0.03%)					
Ileus	2 (0.07%)					
